# Graphdiyne: Recent Achievements in Photo‐ and Electrochemical Conversion

**DOI:** 10.1002/advs.201800959

**Published:** 2018-10-07

**Authors:** Yasong Zhao, Hongjie Tang, Nailiang Yang, Dan Wang

**Affiliations:** ^1^ School of Chemistry and Chemical Engineering Harbin Institute of Technology Harbin 150001 P. R. China; ^2^ State Key Laboratory of Biochemical Engineering CAS Center for Excellence in Nanoscience Institute of Process Engineering Chinese Academy of Sciences No. 1 Beiertiao Zhongguancun Beijing 100190 P. R. China

**Keywords:** 2D materials, carbon materials, electrocatalytic processes, energy‐related processes, photorelated processes

## Abstract

As a rising star of carbon allotropes, graphynes (GYs) merely consist of sp‐ and sp^2^‐hybridized carbon atoms, which endow them a large conjugated network and expanded 2D porous structure. With unique topological structure, GYs display unusual semiconducting properties, especially in the aspects of charge mobility and electron transport. Among the members of the GY family, only graphdiyne (GD) can be successfully synthesized in large quantities. The advanced properties of GD make it promising in various applications. Here, the recent progress in the synthesis of GD and GD‐based composites is reviewed as well as their applications in photorelated and electrocatalytic applications. It is hoped that this Review will promote the development and applications of carbon chemistry.

## 1 Introduction

The growing worldwide energy consumption and the accompanying environmental problem have diverted people's attention on carbon materials, especially the 2D materials, owing to their great advantages in high energy conversion efficiency and environmental friendliness.^1^, ^2^, ^3^, ^4^ Their unique 2D structures with atomic thickness and large surface area result in a high percentage of exposed more active sites and fast carrier transport,^5^, ^6^, ^7^, ^8^ both of which are vital for various energetic and catalytic processes.^9^, ^10^, ^11^, ^12^, ^13^, ^14^, ^15^, ^16^ Graphene, as a representative of carbon allotropes, which is composed of a single layer of sp^2^ carbon atoms packed as 2D honeycomb structure, has drawn extensive attention because of its excellent mechanical, optical and electrical properties as well as high charge carrier mobility.^17^, ^18^, ^19^, ^20^

When the sp^2^ bonds of graphene are substituted by a mix of sp and sp^2^ bonds (i.e., —C≡C—C≡C—), a novel allotrope of carbon is produced, named graphdiyne (GD). Due to the larger diameters of carbon rings in the structure of GD, it is expected to exhibit a better charge and mass mobility as compared with graphene.^21^, ^22^ The density functional theory showed that the intrinsic charge mobility of a single GD layer reaches 2 × 10^5^ cm^2^ V^−1^ s^−1^, comparable with that of graphene (≈3 × 10^5^ cm^2^ V^−1^ s^−1^), indicating its excellent electron transport property.^23^, ^24^ More interestingly, few‐layer GD also exhibits a semiconductor‐like bandgap, which can be diversely tuned by changing the length of chain and size of hexagon, providing great chances for electronics, e.g., field effect transistors.^25^, ^26^

As known, the traditional nanomaterials, such as semiconductors, have their limitation in energy‐related applications due to their poor capability of charge separation and low transition efficiency.^9^ Herein, it is rational to hybridize them with other materials, like GD, to increase the efficiency by making use of the synergistic effect. The GD‐based hybrids have shown their great advantages in achieving the superior performance for the photo‐, and electrochemical applications.^27^ For example, in a certain condition, GD‐semiconductor composite shows excellent electron‐transfer property and a much better performance in photocatalysis compared with graphene‐based composites.^12^, ^17^ Besides, doping heterogeneous atoms in GD can significantly change their inert nature of carbon surface and modify the electronic status, which makes them fascinating for various applications, e.g., lithium ion battery.^28^, ^29^, ^30^, ^31^, ^32^, ^33^, ^34^

Herein, we summarized the methods in preparing 2D GD and GD‐based composites as well as their recent progress in photorelated and electrocatalytic applications. The challenges and prospect of GD are also discussed based on our understanding.

## 2 Synthesis and Properties of GD and Its Composites

In respect to the excellent properties of GD, extensive efforts have been devoted to exploring their synthesis and composition with various functional materials for promising energy‐related applications. We here mainly elaborate their synthetic methods and corresponding properties.

### 2.1 Synthesis of GD and Its Composites

**Figure 1:**
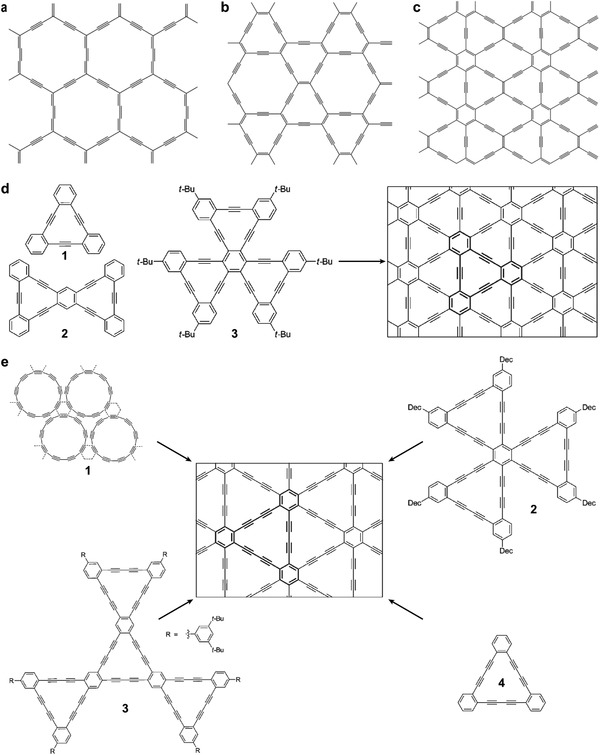
Structures of GYs. a) α‐GY. b) β‐GY. c) 6,6,12‐GY. Reproduced with permission.^36^ Copyright 2003, American Physical Society. d) Organic precursors to synthesize γ‐GY. e) Potential precursors (number 1–4) to construct GD. Adapted with permission.^38^ Copyright 2013, Elsevier.

Graphynes (GYs) were first put forward by Baughman et al. early in 1987, but no detailed investigation has been reported.^35^ Although several kinds of GYs have been predicted (**Figure** **1**a–c),^36^, ^37^, ^38^ there are merely some fragments that can be obtained (Figure 1d).^21^, ^22^, ^23^, ^24^, ^25^ Nevertheless, GYs still attract lots of interest due to their unique structure and properties, such as Dirac points and cones.^26^, ^39^ Their direction‐dependent electronic properties are reflected in unique structures, and the intrinsic mobilities of holes and electrons in 6,6,12‐GY can reach 4.29 × 10^5^ and 5.41 × 10^5^ cm^2^ V^−1^ s^−1^, respectively, which are even larger than that of graphene (≈3 × 10^5^ cm^2^ V^−1^ s^−1^).^40^

GD, a member in GY family that consists of diacetylene groups and benzene ring, was first proposed by Haley,^41^, ^42^ and it is the only GY member which can be synthesized in large scale. It was mentioned that dehydrobenzo annulene framework could lead to the formation of GD and the metal‐catalyzed coupling reaction is necessary to construct the target product (Figure 1e).^38^, ^43^

#### 2.1.1 Synthesis of GD

Although some frameworks were obtained at early time, the self‐standing GD sheets were not successfully synthesized until 2010.^44^ The synthesis of 2D GD films has drawn much attention, and the synthesis of GD with other dimensions and morphology, including 0D nanoparticles, 1D nanowires and 3D sponge, have also been developed.

**Figure 2:**
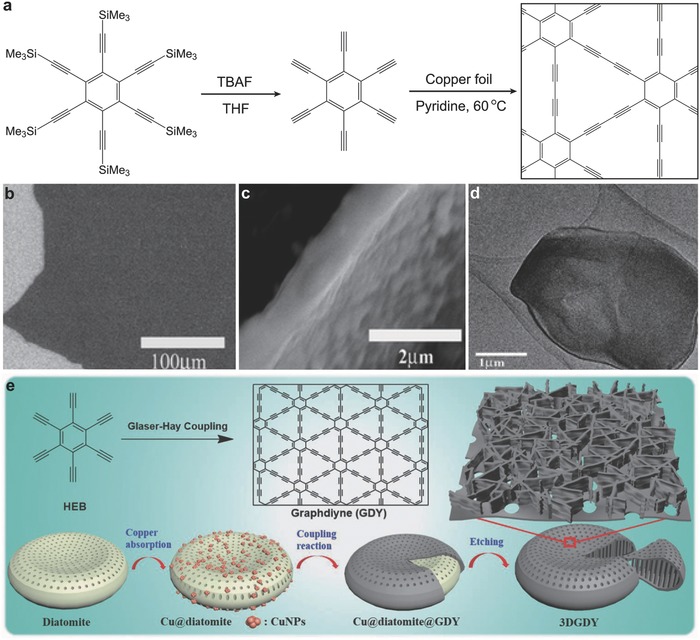
Synthesis of GD. a–d) The synthesis, and scanning electron microscopy (SEM) and TEM images of GD via coupling reaction on the copper foil. Reproduced with permission.^44^ Copyright 2010, The Royal Society of Chemistry. e) Scheme for the synthesis of 3D GD using diatomite as template. Reproduced with permission.^49^ Copyright 2018, Wiley‐VCH.

*Synthesis of 2D GD*: The large‐scale synthesis of 2D GD was achieved via crosscoupling reaction of hexaethynylbenzene (HEB) on the copper foil surface in pyridine through solution polymerization (**Figure** **2**a–d).^44^ Copper was used as both substrate and catalyst to grow GD film. The obtained multilayered GD sheets have a flatten surface with a thickness of ≈1 µm.

To obtain 2D film with larger area, treatments consisting of thermally induced evaporation and crosscoupling reaction process were introduced.^45^ The evolution of GD is dominated by an evaporation process at 200 and 300 °C. When the temperature increased to 400 °C, the GD nanoparticles left the surface to construct uniform and smooth GD films. The growth mechanism is proposed that the heating temperature is the key parameter for evaporating oligomers and triggering the thermal crosscoupling reaction, while the heating time ensures the proceeding of the thermal crosscoupling reaction. By controlling the heat‐treatment process, the large‐area GD films with uniform morphology have been constructed. The GD films possess more lithium storage sites and can be used as electrodes, leading to superior electrochemical performance.

As a variation of GD, TP‐GD (triphenylene as the aromatic core) films were prepared by a liquid/liquid interfacial synthesis method.^46^ Through polymerization, TP‐GD was fabricated as a thin yellow film at the liquid/liquid interface. The resulting free‐standing TP‐GD film presents a smooth surface and its domain size can reach 1 mm with a thickness of 220 nm.

*Synthesis of 0D, 1D, and 3D GD*: 0D GD nanoparticles were obtained by a simple grind‐sonication method.^47^ In detail, 2D GD sheets were manually grounded in an agate mortar and then dispersed in deionized water by tip‐probe ultrasonication with an ice bath overnight. The diameter of the resulted GD nanoparticles was about 10 nm from transmission electron microscopy (TEM) image and 11.16 nm by the dynamic light scattering measurement.

1D GD nanowires were prepared on well‐defined silver surfaces by surface‐confined coupling of specifically designed precursors on the basis of covalent coupling and supramolecular concepts.^48^ Briefly, the precursor compounds were sublimated from quartz glass crucibles inside Knudsen cell at 420 and 460 K, whereas evaporation was carried out onto the substrates which was held below 300 K to guarantee that no covalent reactions occurred. Then the coupling reactions were initiated by thermal annealing the sample. Resultantly, 1D conjugated GD nanowires were fabricated.

3D GD sponge was synthesized from the skeletons of melamine sponge (MS) via a facile in situ Glaser–Hay coupling.^16^ Briefly, macroporous MS was immersed in a tetrahydrofuran solution of HEB to facilitate the absorption of HEB to the MS skeletons by van der Waals forces. Subsequent polymerization of HEB was realized by cuprous iodide‐catalyzed homocoupling of the terminal alkyne units on the surface of the MS. Finally, brown‐colored GD on a melamine sponge substrate was obtained with high flexibility, absorption capacity, and low density.

In addition to sponge, abundant and inexpensive diatomite was also employed as template by Li et al. to construct freestanding 3D GD.^49^ Diatomite was loaded with copper nanoparticles, in which diatomite and copper nanoparticles were used as the substrate and catalyst source, respectively, to make the Glaser–Hay coupling happen. Hollow 3D GD structure was obtained (Figure 2e) after etching out the residual copper and diatomite. The porous structure and high specific surface area of 3D GD sponge enable it to be used as advanced anode material for lithium‐ion battery.

Later, Shang et al. constructed a 3D all‐carbon mechanical and conductive GD networks by a novel in situ weaving strategy on Si anode at ultralow temperature (25 °C).^50^ The 3D GD structures were obtained from the HEB solution by Glaser crosscoupling reaction.

However, since most GD structures are inevitably grown on copper substrates, it is quite complicated to transfer them onto some specific substrates, which limits the application of GD on functional devices. To solve this issue, Zhang and co‐workers reported a feasible route to achieve in situ growth of GD nanowalls on various substrates by enclosing the substrates into a copper foil.^51^ With this method, structure‐controlled GD can be obtained on 1D (Si nanowires), 2D (Au, Ni, W foils, and quartz), and even 3D substrates (stainless steel mesh and graphene foam). Furthermore, an explosion approach was developed by Li et al. for the large‐scale preparation of GD at 120 °C in air without any metal catalyst.^52^ The synthesized GD shows tunable morphology by controlling the heating rate and atmosphere, and the structure can be varied from 1D nanochains to 2D nanoribbons to 3D framework.

### 2.1.2 Synthesis of Few‐Layer GD

Few‐layer GD with larger exposed surface is supposed to possess better electron transfer properties compared with its bulk counterpart.^53^ Therefore, it is meaningful to obtain few‐layer GD in large amount and high quality. Nowadays, there are several methods to achieve thinner GD sheets, such as lithium ion intercalation, oxidized exfoliation, template synthesis, and gas/liquid interface coupling reaction.

**Figure 3:**
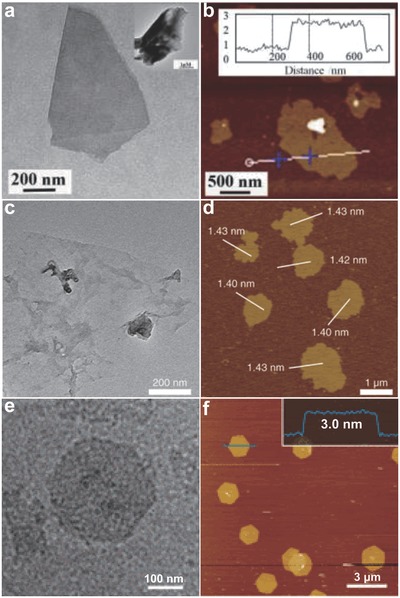
The morphology of few‐layer GD prepared via different methods. a,b) The TEM and atomic force microscopy (AFM) images of few‐layer GD obtained from the lithium ion intercalation method. Reproduced with permission.^54^ Copyright 2017, Wiley‐VCH. c,d) The TEM and AFM images of few‐layer GD obtained by oxidized exfoliation method. Reproduced with permission.^55^ Copyright 2018, Nature Publishing Group. e,f) The TEM and AFM images of few‐layer GD obtained by gas/liquid interface coupling reaction. Reproduced with permission.^58^ Copyright 2017, American Chemical Society.

As for lithium ion intercalation method, GD was used as cathode in a lithium cell and lithium foil was used as the anode. By optimizing the cutoff voltage to induce the intercalation of Li ions between GD layers , well‐dispersed ultrathin GD sheets were obtained after a postsonication treatment (**Figure** **3**a,b).^54^ Besides, strong acid (nitric acid) can also be used as oxidizing and intercalating agent to induce the exfoliation of GD in a controlled manner. After high‐temperature oxidation, washing and centrifugation at different rotating speed, thinner GD nanosheets can be finally obtained (Figure 3c,d).^55^

With respect to template synthesis, graphene can serve as a template to grow ultrathin GD films.^56^ First, graphene was first transferred to SiO_2_/Si substrate. After immersing the substrate into precursor solution, ultrathin GD film was obtained via controlled polymerized growth in the presence of CuCl catalyst. The thickness is varied from 3 to 20 nm by controlling the concentration of monomer. Li et al. also prepared an ultrathin β‐GD‐like film by Eglinton coupling reaction through template synthesis due to the strong π–π interaction between β‐GD and graphene.^57^

Previous methods cannot avoid the random encounter of HEB precursor and the catalysts, leading to the inevitable formation of GD nanoparticles besides nanosheets. To overcome this disadvantage, interfacial polymerization method has been developed.^58^ A thin GD nanosheet was finally synthesized at the interfaces of water and the selected organic solvents (Figure 3e,f).

### 2.1.3 Preparation of GD‐Based Composite

Recently, numerous efforts have been devoted to the design, fabrication, and modification of various GD‐based functional composites for energy conversion and environmental protection. The hybrid materials can synergistically take advantage of each component to further boost the overall catalytic activity. In the following parts we will introduce the synthesis methods in detail with classification based on the dimensions of applied materials, such as element‐hybrid (doping) and material‐hybrid (0D/GD, 1D/GD, and 2D/GD). The synthetic methods mainly involve simple solution mixing, hydrothermal reaction, oxidation‐reduction, impregnation, ion‐beam sputtering, and the thermal pyrolysis method.

The meaning of functionalization of these 2D carbon materials is significant. For photochemistry in semiconductors, when the electron in the valance band (VB) is excited into conduction band (CB), a hole is generated in the VB. However, the generated holes and electrons in excited states are not stable and they are easy to recombine. Thus, designing photocatalysts to suppress the recombination of holes and electrons and improve photocatalysis performance is highly desired. One common strategy is to produce hybrid photocatalyst by loading semiconductors on various carbon substrates. Due to the stronger electron affinity and transfer capability, the recently emerged 2D carbon allotropy, namely GD, is considered as a better assistant in photocatalysis compared with other carbon substrates. Besides, most surfaces of carbon materials are chemically inert, which need further modification to optimize the electronic properties for electrochemistry. Doping with other light elements can effectively break the electron neutrality and promote the intrinsic catalytic activity. In this section, we summarize the recent development in the preparation of various GD‐based composites for photo‐ and electrochemical conversion.

**Figure 4:**
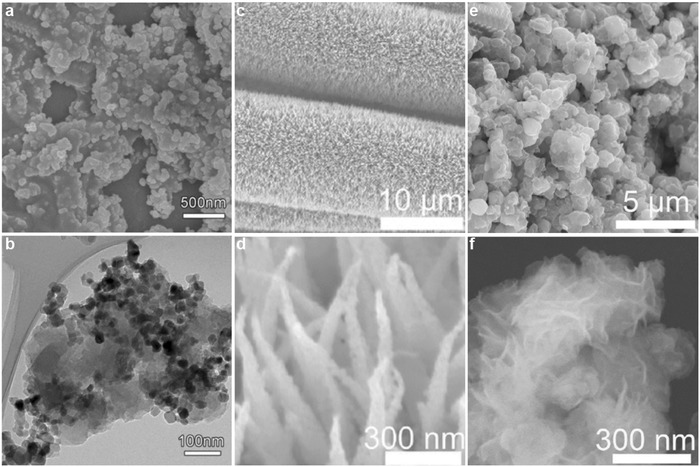
The morphology of hybrid catalysts. a) SEM image and b) TEM image of 0D P25 nanoparticles/GD composite catalysts. Reproduced with permission.^60^ Copyright 2012, Wiley‐VCH. c) SEM image and d) TEM image of 1D NiCo_2_S_4_ nanowires/GD composite catalysts. Reproduced with permission.^66^ Copyright 2017, Wiley‐VCH. e) SEM image and f) TEM image of 2D MoS_2_ nanosheets/GD composite catalysts. Reproduced with permission.^67^ Copyright 2018, Wiley‐VCH.

*0D/GD Hybrid*: Extensive efforts have been devoted to developing highly efficient and stable catalysts by compositing various 0D metals or semiconductors with GD, such as Ag/AgBr/graphene oxide (GO)/GD, P25‐GD, TiO_2_‐GD, Pd/GD, PtNP‐graphdiyne nanosheet (GDNS), Fe/GD, and Pt‐GD. For example, Zhang et al. prepared Ag/AgBr/GO/GD by mixing the chloroform solution of Ag precursor (AgNO_3_) with GO/GD suspension by an ultrasonic homogenizer.^59^ As another example, with a facile one‐pot hydrothermal method, Wang et al. successfully synthesized P25‐GD nanocomposite photocatalyst (**Figure** **4**a,b).^60^ It has been demonstrated that the large delocalized π system of GD can anchor TiO_2_ nanoparticles by forming a strong chemical bond of Ti‐C. Based on that, Yang et al. synthesized TiO_2_ (001)‐GD by a simple hydrothermal method with the perfect interaction between the crystalline TiO_2_ nanosheets and GD.^61^

Besides as the template, GD can serve as both reductant and stabilizer for direct synthesis of ultrafine Pd nanoparticles (NPs).^62^ In addition, GD was also successfully utilized as substrate to disperse single atoms. Xue et al. and Yin et al. successfully prepared single atoms of Ni, Fe and Pt on GD though a simple impregnation method to further increase atom utilization.^63^, ^64^

Moreover, some physical techniques have also been adopted to fabricate the 0D/2D hybrid. For example, Ren et al. has reported the ion‐beam sputtering approach to deposit Pt nanoparticles onto pre‐exfoliated GD substrates.^65^

*1D/GD Hybrid*: The fabrication of 1D/GD hybrid materials is rarely reported. One successful example is vertically growing NiCo_2_S_4_ nanowires on the surface of 3D GD scaffolds (Figure 4c,d).^66^ The rough GD surface with uniformly distributed pores can provide a large catalytically active surface area and facile contact with electrolyte, making it a highly active and stable 3D bifunctional electrocatalyst for overall water splitting.

*2D/GD Hybrid*: The combination of different 2D materials can generate good catalysts for advanced photo‐ and electrochemical conversion. Up to date, GD has been composited with various 2D materials, such as transition metal disulfides, layered double hydroxide (LDH), other carbon materials and even metal composites, to achieve better catalytic performance.

Yu et al. reported that the nitrogen‐doped GD (NGD) with porous structure and excellent conductive network can be used as the robust support for the growth of MoS_2_ nanosheets (Figure 4e,f).^67^ By a facial solvothermal method, in which NGD, Na_2_MoO_4_ • 2H_2_O and thiourea were mixed and the temperature was maintained at 200 °C for 20 h, MoS_2_ nanosheets were in situ grown on NGD. The heterostructure with strong interaction between NGD and MoS_2_ endows them fast charge transport and is favored for hydrogen evolution reaction (HER). Through the similar solvothermal method, Han et al. prepared a metal‐free 2D/2D heterojunction composed of GD and g‐C_3_N_4_.^68^

Hui et al. introduced porous GD nanolayers on the surface of iron carbonate hydroxide (FeCH).^69^ The FeCH was first coated onto Ni foams by hydrothermal method and the resulted brown‐colored FeCH/nickel foam (NF) was further wrapped by ultrathin GD.

*Doping*: Besides the hybridization with different materials, doping is another effective method to enhance the catalytic activity of GD. N atom is one of the commonly reported doping atoms. The N atoms could be doped into the carbon structures, existing as three common bonding forms: pyridinic N, pyrrolic N, and graphitic/quaternary N.^70^ The function of the different kinds of doped nitrogen atoms has been discussed extensively, but the active sites for oxygen reduction reaction (ORR) still remain unclear.^71^

The most efficient and well‐studied NGD catalysts can be obtained by reacting GD with various N‐containing precursors at high temperature, such as aniline, NH_3_, and pyridine. For example, nitrogen‐containing carbon nanolayers on GD nanostructure were synthesized by one‐step pyrolysis of iron salts and polyaniline loaded onto GD nanocomposite.^72^ Liu et al. prepared NGD by annealing GD under ammonia in tube furnace. By carefully optimizing the heating procedures, N atoms are successfully incorporated into GD structures.^73^ In order to further increase the N content, multiple nitrogen sources can also be introduced. The mixture of GD and pyridine was treated at high temperature to obtain NGD and further calcination under NH_3_ atmosphere can result in the formation of N′N‐GD.^74^ The interfacial synthesis has also been employed to prepare NGD. Pan et al. synthesized NGD nanosheets containing different number of N via polymerization of triazine, pyrazine, and pyridine‐based monomers at liquid/liquid interface.^75^

The bottom‐up synthetic strategy can also be used to obtain GD doped with other heteroatoms. For example, Wang et al. synthesized boron‐GD (BGD) and chlorine‐substituted GD (Cl‐GD) in the presence of 1,2,3‐trichlorineborane and 1,3,5‐trichlorinebenzene by Glaser–Hay coupling reaction.^7^, ^12^ The boron and chlorine heteroatoms are evenly distributed in the BGD and Cl‐GD framework with precise control.

## 2.2 Properties

### 2.2.1 Electronic Properties

**Figure 5:**
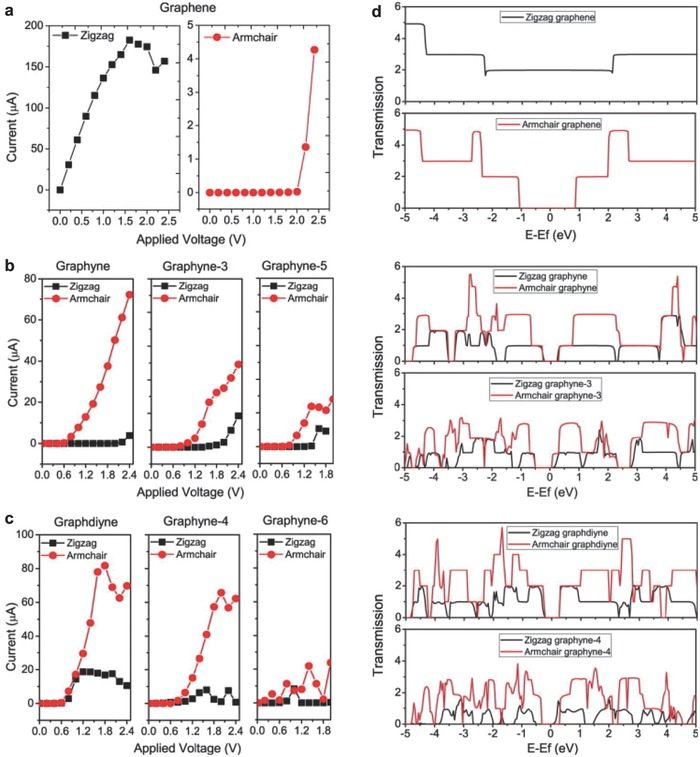
The *I–V* curves and total transmission spectra of graphene and GYs. a) *I–V* curves of graphene. b) *I–V* curves of GY, GY‐3, and GY‐5. c) *I–V* curves of GD, GY‐4, and GY‐6. d) The total transmission spectra of graphene and GYs. Reproduced with permission.^76^ Copyright 2013, Elsevier.

It is necessary to analyze the electronic properties for in‐depth understanding of GYs. The nonequilibrium Green's function method and density functional theory (DFT) are used to study the electron transport properties of GYs. GYs display a semi‐conductive characteristic along zigzag direction and metallic characteristic along armchair direction, which differs a lot from graphene.^76^ The electron transport properties of GYs are associated with the length of C link in their structures. With the length of C link increasing, their electrical conductivity decreases. Besides, graphene and GYs present different *I–V* curves in different current transport directions (**Figure** **5**a–c). The *I–V* curves of GYs show regular changes with the increasing C amount of the C atomic links (denoted as GY‐*n*). GY, GY‐3, and GY‐5 show similar transport properties (Figure 5b), while GD, GY‐4, and GY‐6 display similar transport properties (Figure 5c). As the number of acetylene bond increases, and the height of the transmission spectrum around Fermi energy decreases, which lead to the decreased conductivity of GYs (Figure 5d).

For bulk GD, its electronic properties are also investigated using DFT, which are associated with its stacking configurations.^77^ At the Heyd–Scuseria–Ernzerh (HSE06) level, the AA configuration is a metal, and the AB‐1, AB‐2, and AB‐3 configurations are semiconductors with bandgaps of 0.05, 0.74, and 0.35 eV, respectively. For a single GD sheet, the electron mobility can reach 2 × 10^5^ cm^2^ V^−1^ s^−1^) at room temperature, while its hole mobility is an order of magnitude lower.^23^

As for GD nanoribbons (GDNRs), the electron mobility at room temperature is about 10^4^ cm^2^ V^−1^ s^−1^), which is also greatly larger than that of the hole mobility. It is found that the charge mobility increases with the width of GDNRs, and the divan‐edged GDNRs have larger mobility than the zigzag edged GDNRs. In addition, both the zigzag and armchair GDNRs display semi‐conductive characteristic.^76^

Bilayer GD (AB style) possesses a direct bandgap of 0.35 eV, whereas trilayer GD (ABA style) has a bandgap of 0.18–0.33 eV. Moreover, the bandgaps of the semiconducting bilayer and trilayer GD generally decrease with increasing external vertical electric field, irrespective of the stacking style.^34^

### 2.2.2 Mechanical Properties

The elastic properties (in‐plane stiffness and Young's modulus) of GD are studied using a self‐consistent charge density functional tight‐binding method. The results indicate that as the length of the introduced acetylene links in GD increases in the *x*‐direction, the in‐plane stiffness and Young's modulus increase and finally approach constant values.^78^ Hou et al. proposed that the in‐plane stiffness is a decreasing function whereas Poisson's ratio is an increasing function of the number of acetylenic linkages between two adjacent hexagons in GY‐*n*.^79^ GD is a softer material than graphene, with an in‐plane stiffness of 120 N m^−1^, which is equivalent to a Young's modulus of 375 GPa if a thickness of 0.320 nm is assumed.^80^ Pei et al. predicted the in‐plane stiffness and calculated the Poisson's ratio as 0.453 by performing ab initio calculations using the Vienna ab initio simulation package.^81^

Mechanical properties of GYs with different number of acetylenic linkages are also investigated by molecular dynamics simulations. Yang et al. found that in the armchair loading case, the fracture strain remains nearly unchanged whereas the ultimate strength degrades gradually with longer acetylenic chains.^82^ In the zigzag loading situation, the ultimate strength remains nearly the same whereas the fracture strain improves by a little amount with longer acetylenic chains.

### 2.2.3 Optical Properties

Luo et al. investigated the optical properties of several possible GD bulk structures.^77^ It was found that bulk GD can be either a semiconductor or a metal, depending on its stacking configuration. The interlayer van der Waals force redshifts the optical absorption peaks of bulk GD relative to those of the monolayer, and the spectra of different stacking modes display obvious differences in the energy range below 1 eV.

First principle calculations with generalized gradient approximation were used to analyze the optical properties of armchair and zigzag GY nanotubes (GNTs) doped with boron (B) and nitrogen (N) atoms.^83^ The resulting bandgap tuning was investigated with respect to the B/N substitution site and increasing diameter of the NTs. Dependence of the absorption coefficient, optical conductivity, reflectivity, and refractive index on the tube diameter has been found. The origin of the optical responses was monitored from the infrared to the UV region depending on the doping site of the B/N. For B, N doped GNTs, the entire absorption spectrum lies in the UV region, showing great possibility of usage for UV light protection or short wavelength optoelectronic devices.

## 3 Photorelated Applications

The efficient interfaces constructed between GD and semiconductors can result in enhanced charge transfer and improved performance. In this case, GD and its composites have been widely utilized in photocatalysis, solar cells, and electrocatalytic reactions.

### 3.1 Photocatalytic Applications

Semiconductor photocatalysts have drawn much attention in environmental remediation and their charge generation, separation and transport efficiency determine the overall performance.^84^, ^85^, ^86^ The easy recombination of photogenerated electrons and holes greatly limits the efficiency of photocatalytic applications. To suppress the recombination of charge carriers and enhance photocatalytic activity, hybrids of the semiconductor and carbon material have attracted considerable attention.

In this part, three main applications of GD‐based composites are reviewed, including photocatalytic degradation, photocatalytic reduction, and photocatalytic hydrogen generation.

#### 3.1.1 Photocatalytic Degradation

In recent years, photocatalytic degradation plays an increasingly important role in dealing with environmental problems, such as water purification, air cleanup, and waste remediation. Photogenerated electrons and holes of separate semiconductors, especially TiO_2_, are easy to recombine. GD, as a novel form of carbon materials, possesses many exceptional properties, including high surface area and high electron mobility. GD‐based semiconductor photocatalysts can embrace synergistic effects between the metal oxides and carbon materials, which widen light absorption range and suppress electron–hole recombination.^60^

**Figure 6:**
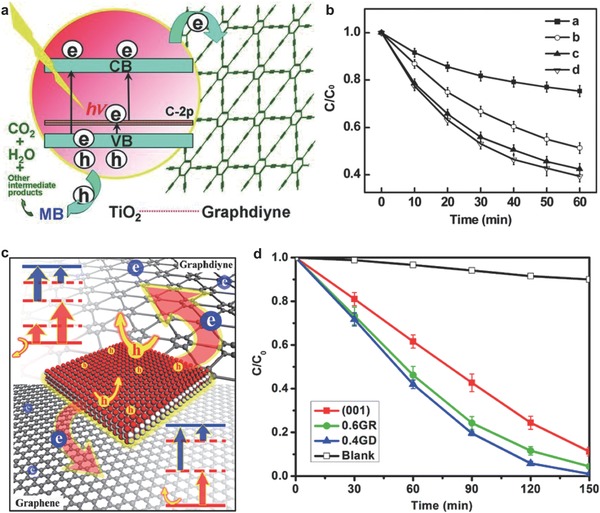
Schematic diagram and photocatalytic performance of GD. a) Schematic structure of photodegradation processes of MB using P25‐GD as the catalyst. b) Photocatalytic degradation of MB under visible light with a–d representing P25, P25‐CNTs, P25‐GR, and P25‐GD, respectively. Reproduced with permission.^60^ Copyright 2012, Wiley‐VCH. c) Schematic diagram of the electronic structure of 2D carbon‐supported TiO_2_. d) Photocatalytic degradation of MB with TiO_2_ (001), TiO_2_ (001)‐GD, TiO_2_ (001)‐GR catalysts, and the blank as control group. Reproduced with permission.^61^ Copyright 2013, the American Chemical Society.

As reported before, TiO_2_ has attracted much attention in photocatalytic degradation of contaminants due to strong UV light absorption and photostability. Wang et al. reported a hybrid of commercialized TiO_2_ (P25) and GD for photocatalytic degradation of organic dyes (**Figure** **6**a).^60^ The hydrothermal treatment method makes the P25 nanoparticles chemically bonded with GD, forming Ti—O—C bond. The impurity bands introduced by carbon p orbitals make P25‐GD exhibit a visible‐light photocatalytic activity. The resulting P25‐GD composite presents an obvious enhancement for degrading methylene blue (MB) compared with P25, P25‐carbon nanotubes (P25‐CNTs), and P25‐graphene (P25‐GR). The photocatalytic degradation efficiency of MB under visible light follows the order of P25‐GD > P25‐GR > P25‐CNTs > bare P25. In particular, the photocatalytic activity of P25‐GD was increased by 4.5% compared to that of P25‐GR (Figure 6b). During this process, GD served as an acceptor for the photogenerated electrons, which helps suppress the charge recombination, thus facilitating the degradation of dyes.

To further understand the charge transfer mechanism, it is necessary to explore the interactions between the crystal facets of TiO_2_ and GD. By using DFT to calculate the electronic properties between GD and TiO_2_ with different crystal facets, Yang et al. first demonstrated that TiO_2_ (001)‐GD possessed the highest charge separation capability, the strongest oxidation ability, and the most abundant impurity levels (Figure 6c).^61^ In detail, the GD has the strongest bonding with (001) facet of TiO_2_, which can effectively suppress the charge recombination and promote the charge separation, and lifetime of the photoexcited carriers of TiO_2_ (001)‐GD also remains longer than that of TiO_2_ (001)‐GR. The VB position of TiO_2_ (001)‐GD is lower than that of TiO_2_, indicating its higher oxidation ability. In addition, TiO_2_ (001)‐GD possesses positive and negative charges, and GD can introduce impurity levels to TiO_2_ more easily than graphene, resulting in abundant impurity levels. The photocatalytic degradation of MB is shown in Figure 6d. For the pure TiO_2_ (001), the first‐order rate constant *k* is estimated to be 0.0152 min^−1^ by normalizing temporal concentration change (*C*/*C*_0_) of MB with respect to time, whereas TiO_2_ (001)‐GR composite with 0.6 wt% GR shows a 1.28 times improvement (0.0195 min^−1^). Interestingly, for TiO_2_ (001)‐GD composite with 0.4 wt% GD, *k* is up to 0.0247 min^−1^, which is 1.62 times that of pure TiO_2_ (001) and the highest among all these TiO_2_ (001)‐based catalysts.

To further increase the photocatalytic activity, ZnO nanoparticles were introduced because their electron mobility is higher than that of TiO_2_ by two orders of magnitude. Thangavel et al. synthesized the hybrid of ZnO‐GD via a hydrothermal method.^87^ The result presented that ZnO‐GD is more efficient than bare ZnO NPs in the photodegradation of MB and rhodamine B, and the rate constant of photodegradation for ZnO‐GD nanohybrids is almost twofold higher than bare ZnO.

#### 3.1.2 Photocatalytic Reduction

Since GD has lower reduction potential, it can be used as a reactant to reduce metal ions. Qi et al. reported that the GD has strong interaction with Pd, which can dramatically avoid the aggregation of atom, and serve as a substrate to deposit ultrafine Pd clusters.^62^ The synergistic effects between highly dispersed Pd clusters and large π‐conjugated network of GD contributed to the successful reduction of 4‐nitrophenol (4‐NP). When Pd/GD was added into the 4‐NP, the absorption peak at 400 nm decreased obviously and a new peak emerged at 300 nm, demonstrating the reduction of 4‐NP. Among the four kinds of catalysts, i.e., Pd/CNT, Pd/GO, Pd/C, and Pd/GD, Pd/GD possessed the highest catalytic activity.

In addition, N doping can make the catalyst hydrophilic, and the resulting NGD could be employed as metal‐free photocatalyst.^75^ This approach provided a new road for designing catalysts.

#### 3.1.3 Photocatalytic Hydrogen Generation

Hydrogen energy, as a powerful and clean fuel source, has attracted great attention. Photoelectrochemical hydrogen production by semiconductor is regarded as a promising means in future.

**Figure 7:**
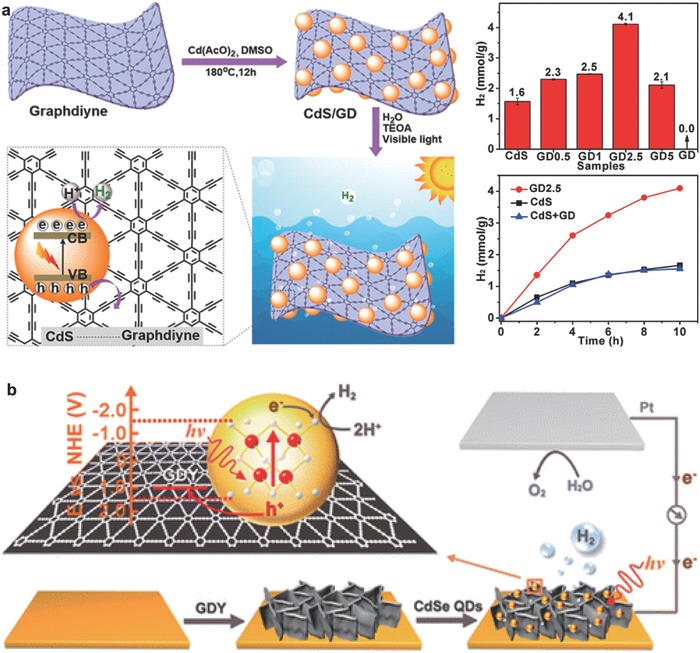
Synthesis and photocatalytic activity of GD‐based composites. a) Synthesis, photocatalytic process of the hybrid CdS/GD, and its photocatalytic performance. Reproduced with permission.^91^ Copyright 2018, American Chemical Society. b) Synthesis of CdSe QDs and the corresponding photogenerated excitons process. Reproduced with permission.^92^ Copyright 2016, American Chemical Society.

Metal chalcogenides, especially CdS nanoparticles, have obtained much attention due to relatively negative CB position.^88^, ^89^, ^90^ However, the poor structure stability and severe recombination of charge carriers during the photocatalytic process limit the applications of CdS nanoparticles. Lv et al. successfully prepared CdS/GD heterojunction by in situ growth process, which exhibited good performance for photocatalytic hydrogen evolution (**Figure** **7**a).^91^ The mass activity of the composite catalysts increased with GD content, and GD2.5 possessed the highest activity (4.1 mmol g^−1^), which is 2.6 times of pure CdS (1.6 mmol g^−1^). In addition, the photocatalytic activity of GD2.5 is also much higher than that of physical mixture of GD and CdS, indicating the great impact of forming CdS/GD heterojunction on improvement of the photocatalytic activity of CdS.

Among the various structures of semiconductor catalysts, quantum dots (QDs) have been used as an alternative due to exceptional light‐harvesting ability and relatively large surface area. Li et al. prepared CdSe QDs/GD photocathode for the first time, where GD performs as the hole transfer layer (Figure 7b).^92^ The strong interactions between GD and CdSe QDs facilitated hole transportation and improved the performance of hydrogen generation. The optimal CdSe QDs/GD photocathodes exhibited nearly (90 ± 5)% faradic efficiency for 12 h in the hydrogen production tested under Xe lamp.

### 3.2 Solar Cells

Due to the unique electronic property, GD has been widely used in solar cells. The insertion of GD into solar cells can facilitate charge extraction and transport. Generally, GD can play three kinds of roles in solar cells, including counter electrode, electron transport layer, and hole‐transporting layer.

#### 3.2.1 Counter Electrode

As an alternate to silicon‐based solar cells, dye‐sensitized solar cells (DSSCs) have been extensively studied due to its low cost, high efficiency and easy operation, in which the counter electrode is the key part. The requirement for counter electrode in DSSC is that it should possess a higher catalytic activity in catalyzing the triiodide/iodide (I_3_^−^/I^−^) redox couple of the electrolyte.^93^, ^94^, ^95^ Pt has always been employed as the counter electrode owing to its high conductivity and efficient iodine reduction, but its high cost limit the wide application. To reduce the cost of DSSCs, GD was selected as the alternative to Pt.

**Figure 8:**
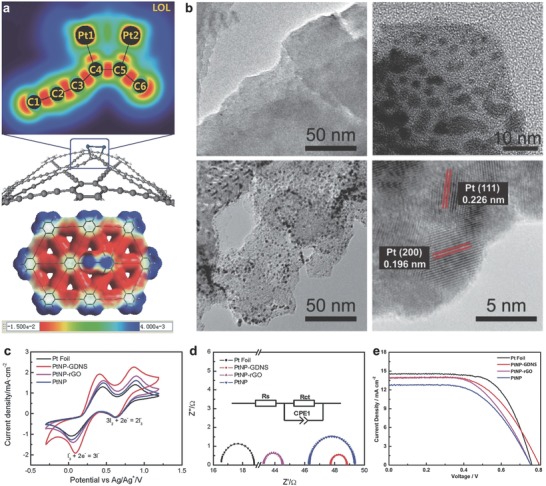
The morphology and performance of Pt nanoparticles/GD as counter electrode. a) Localized orbital locator maps and electrostatic potential (ESP) surfaces. b) TEM and high‐resolution transmission electron microscopy images of Pt nanoparticles. c) Cyclic voltammetry curves of various counter electrodes. d) Nyquist plots of various counter electrodes. e) Photocurrent density–voltage curves of DSSCs on various counter electrodes. Reproduced with permission.^65^ Copyright 2015, Wiley‐VCH.

Layered nanomaterials have attracted much attention due to their larger surface area and more exposed sites compared with bulk materials. In this case, the ultrathin GD nanosheet was used for the counter electrode. Ren et al. first prepared thin‐layer GD nanosheet by using lithium‐ion intercalation method.^65^ The obtained GD nanosheet served as the substrate to deposit Pt nanoparticles by ion‐beam sputtering method, and the interaction between them enhanced the charge transfer from GD to Pt (**Figure** **8**a,b). It is the first time that GD hybrid was used as the counter electrode for DSSCs. Pt‐GD hybrid counter electrode exhibited excellent performance comparable to that of precious Pt foil, and even better than DSSCs with Pt nanoparticles or Pt nanoparticle‐reduced graphene oxide hybrid (Figure 8c–e).

#### 3.2.2 Electron Transport Layer

**Figure 9:**
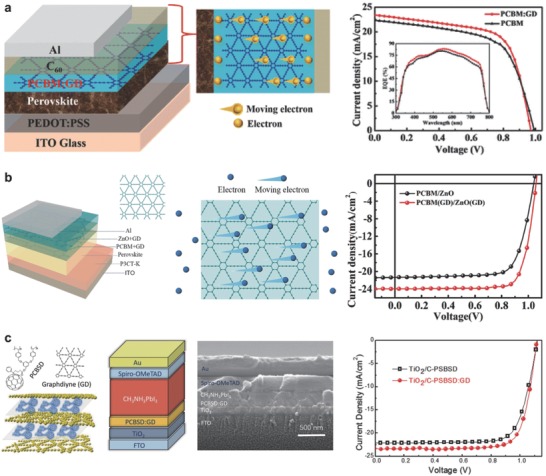
Structures and performance of perovskite solar cells. a) Structure of perovskite solar cell (Al/C_60_/PCBM:GD/perovskite/poly(3,4 ethylenedioxythiophene):poly(styrenesulfonate)/indium tin oxide glass), and the *J–V* curves of perovskite solar cells under AM 1.5G 100 mW cm^−2^ simulated solar light. Inset is the corresponding external quantum efficiency spectra. Reproduced with permission.^96^ Copyright 2015, American Chemical Society. b) The structure of perovskite solar cell, and corresponding *J–V* curves of perovskite solar cells under AM 1.5 G 100 mW cm^−2^ simulated solar light. Reproduced with permission.^97^ Copyright 2018, Elsevier. c) Device structure of solar cells, and *J–V* curves of perovskite solar cells. Reproduced with permission.^98^ Copyright 2018, Elsevier.

High charge carrier mobility and absorption coefficient make organic–inorganic hybrid perovskite solar cell a promising future device. However, problems of low coverage, current leakage, and interfacial recombination limit wide application. An efficient approach is to modify the electron transport layer. GD, as an electron transport layer, shows an increase in electron mobility, electrical conductivity, and charge extraction ability due to unique electronic and optic properties. By introducing GD into perovskite solar cell, the *J*_sc_ increased from 22.3 to 23.4 mA cm^−2^, and the photo conversion efficiency (PCE) increased from 13.5% to 14.8% (**Figure** **9**a).^96^

To further enhance the performance, a more effective method is the dual doping GD in both phenyl‐C_61_‐butyric acid methyl ester (PCBM) and ZnO films of perovskite solar cells (Figure 9b).^97^ This approach led to an enhancement of PCE from 16.6% to 20.0% based on MAPbI_3_ active layer. Li et al. adopted GD‐modified crosslinkable fullerene in organometal halide as an electron transport layer, which also increased the performance of perovskite solar cells (Figure 9c).^98^

#### 3.2.3 Hole‐Transporting Layer

The hole‐transporting material (HTM) layer is critical for improvement of perovskite solar cells. Doping GD into HTM can greatly hinder the recombination of generated electrons and holes and improve charge extraction and transport. With this method, Xiao et al. demonstrated the hybrid of poly (3‐hexylthiophene) (P3HT) and GD can increase the cell performance owing to the formation of strong π–π stacking interaction.^99^ The light‐to‐electricity conversion efficiency was up to 14.58%, much better than that of P3HT‐based devices. Also, Li et al. introduced GD into P3CT‑K of MAPbI_3_ perovskite solar cells, which served as an efficient hole‐transport layer, and a high efficiency of 19.5% was achieved.^100^

## 4 Electrocatalytic Applications

### 4.1 Oxygen Reduction Reaction

With great efforts being devoted into studying non‐noble metal ORR catalysts, a novel class of metal‐free ORR catalysts has recently attracted researcher's attention.^101^ Carbon materials, as alternative catalysts, hugely reduce the cost of fuel cells.^102^, ^103^ Heteroatom‐doped carbon structures, e.g., N‐doped graphite, N‐doped graphene, and N‐doped nanotube, exhibit excellent catalytic activity.^101^, ^104^, ^105^, ^106^, ^107^, ^108^, ^109^, ^110^, ^111^ GD, as one of the carbon allotropes, can also serve as ORR catalysts, because of the existence of some positively charged carbon atoms induced by the introduction of nitrogen atom.^112^, ^113^ The electronic structure of GD can also be modulated after doping, endowing GD with a higher electrochemical activity.

**Figure 10:**
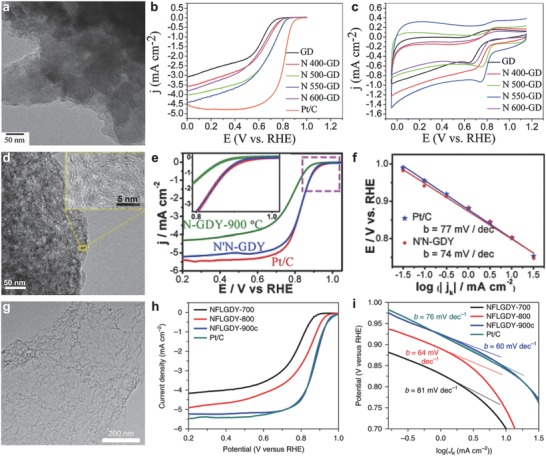
ORR performance in alkaline solution. a) TEM image of N 550‐GD. b,c) Linear sweep voltammetry (LSV) and cyclic voltammogram (CV) curves of GD, NGD, and Pt/C in an O_2_‐saturated 0.1 m KOH solution. Reproduced with permission.^73^ Copyright 2014, The Royal Society of Chemistry. d) TEM image of N′N‐GD. e) LSV curves of N‐GD, N′N‐GD, and Pt/C in O_2_‐saturated 0.1 m KOH. f) Tafel plots of N′N‐GD and Pt/C. Reproduced with permission.^74^ Copyright 2017, American Chemical Society. g) TEM image of NGD‐900. h) LSV curves of NGD‐700, NGD‐800, NGD‐900c, and Pt/C in O_2_‐saturated 0.1 m KOH solution. i) Tafel plots of NGD‐700, NGD‐800, NGD‐900c, and Pt/C. Reproduced with permission.^55^ Copyright 2018, Nature Publishing Group.

Zhang et al. prepared NGD using high‐purity ammonia.^73^, ^114^ Although the NGD catalyst displayed better stability and tolerance to methanol than commercial Pt/C catalysts, the ORR catalytic activity is still very low (**Figure** **10**a–c). To further improve the ORR activity, Lv et al. conducted the doping of N element twice, using pyridine and ammonia as nitrogen source, respectively. The resulting NGD exhibited an activity that is similar to that of commercial Pt/C and higher than previously reported catalysts (Figure 10d–f).^74^

Further improvement of the ORR activity of GD‐based catalysts still remains a challenge. Besides regulating the nitrogen amount to improve the activity, the forms of doped nitrogen can be also adjusted, which might have more positive effects on the performance. In view of the unique structure of GD, the high‐energy acetylenic linkages make it possible to introduce the sp‐hybridized N through pericyclic reactions. Zhao et al. prepared NGD via pericyclic reaction, using melamine as the nitrogen source and few‐layered GD as the carbon source.^55^ The nitrogen content can be tuned by the amount of melamine, and the nitrogen forms appear as sp‐hybridized N, pyridinic N, amino N, and graphitic N. The as‐prepared sp‐N‐doped GD catalyst presented excellent ORR performance with *E*_1/2_ of 0.87 V and *J*_k_ of 38.0 mA cm^−2^ at 0.75 V, even surpassing commercial Pt/C under alkaline conditions, and it showed comparable performance in acidic media (Figure 10g–i). DFT calculations also suggest that the high catalytic activity is originated from sp‐hybridized N atoms.

### 4.2 Oxygen Evolution Reaction (OER)

ORR is vital to fuel cell. Likewise, OER also plays an important role in metal–air batteries and water splitting devices. Over the past decades, the OER catalysts are mainly limited to noble metal oxides, such as iridium and ruthenium oxides, which hinders the large‐scale commercialization.^115^, ^116^, ^117^

**Figure 11:**
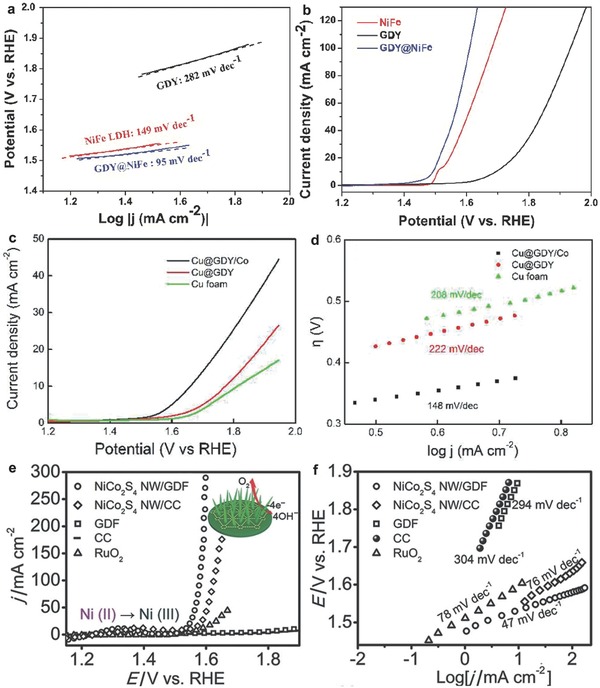
OER performance in KOH solution. a) Tafel slopes and b) LSV curves of GD, NiFe LDH, and GD@NiFe composite catalysts. Reproduced with permission.^118^ Copyright 2018, American Chemical Society. c) LSV curves and d) the corresponding Tafel slopes of Cu foam, Cu@GD, and Cu@GD/Co catalysts. Reproduced with permission.^119^ Copyright 2017, American Chemical Society. e) LSV curves and f) the corresponding Tafel slopes of NiCo_2_S_4_ nanowire (NW)/GD, NiCo_2_S_4_ NW/carbon cloth (CC), GD, CC, and RuO_2_. Reproduced with permission.^66^ Copyright 2017, Wiley‐VCH.

Transition metal LDHs are always considered as promising catalysts for OER. However, the relative low conductivity of LDHs limits the improvement of OER catalytic activity. Shi et al. integrated GD and NiFe LDH to synthesize a novel electrocatalyst, and the obtained GD@NiFe composite presented high activity toward OER due to the strong interfacial interaction (**Figure** **11**a,b).^118^

Besides, most electrocatalysts are stabilized on electrodes using polymer binders, such as nafion, which could generate the series resistance and further block active sites, thus reducing catalytic activity. To overcome these issues, it is rational to integrate metal‐based materials with freestanding carbon substrates. These composites can directly serve as electrodes to work in electrolytic solution. In view of the strong interaction between metal ions and alkyne π‐conjugated structures, Li et al. prepared Cu@GD/Co self‐supported electrode, which showed high OER catalytic activity with a small overpotential (Figure 11c,d).^119^

Xue et al. also prepared NiCo_2_S_4_ nanowire/GD catalysts, in which the 3D GD foam served as scaffolds (Figure 11e,f).^66^ The 3D porous and conductive scaffolds can facilitate catalysts to contact with electrolyte, enabling the catalysts to exhibit excellent catalytic activity and cycling stability for OER and HER.

### 4.3 Hydrogen Evolution Reaction

Besides ORR and OER, HER also plays an important role in metal–air cells. Among the catalysts, metal nanoparticles usually have weak interaction with substrates, thus they tend to aggregate, leading to the degradation in HER performance. To solve this problem, it is necessary to strengthen interaction between metal nanoparticles and substrates. The strong interaction between Pt nanoparticles and GD prevents the migration of Pt nanoparticles on GD surface. The composite catalyst loaded with Pt nanoparticles (size: 2–3 nm) showed a high performance for HER.^120^

**Figure 12:**
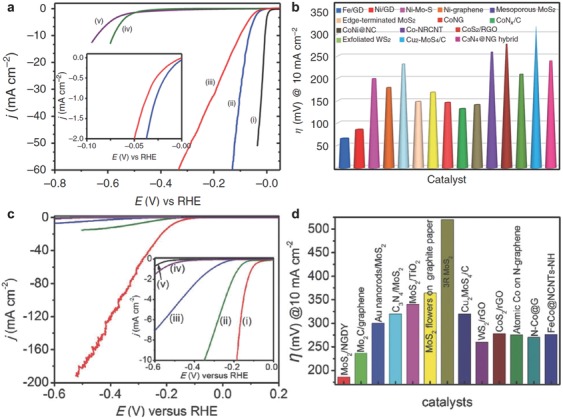
HER performance in acidic solution. a) LSV curves of i) Pt/C, ii) Fe/GD, iii) Ni/GD, iv) graphdiyne foam, and v) CC. b) Overpotentials at 10 mA cm^−2^. Reproduced with permission.^63^ Copyright 2018, Nature Publishing Group. c) LSV curves of MoS_2_/NGD, MoS_2_/GD, MoS_2_, NGD, and GD. d) Overpotentials obtained at 10 mA cm^−2^ of various reported catalysts. Reproduced with permission.^67^ Copyright 2018, Wiley‐VCH.

In order to further increase the atom utilization efficiency, single‐atom catalysts are explored. The isolated nickel/iron atoms were anchored on GD successfully.^63^ Such atomic catalysts showed high catalytic activity and stability for HER (**Figure** **12**a,b).

Besides the noble metals, the hybrid of GD nanosheets and MoS_2_ also showed a high activity and extraordinary stability (Figure 12c,d).^67^ Yao et al. also synthesized layered 2D‐nano‐hybrid GD‐WS_2_ catalyst, showing an onset potential of ≈140 mV and Tafel slope of ≈54 mV dec^−1^ in HER.^121^

## 5 Summary and Outlook

To summarize, this review provides an overview of the recent achievement in the synthesis, properties and up‐to‐date applications of GD and GD‐based composites in photorelated and electrocatalytic applications. This new carbon allotrope, i.e., GD, has excellent intrinsic electrical, mechanical, and optical properties. When combined with metal nanoparticles, semiconductors or doped with other elements, it can greatly enhance the charge separation and transportation during the photorelated or electrocatalytic conversion processes, bridging the gaps between semiconductors or metal nanoparticles by providing good electrical contact and low interfacial resistance. Their excellent performance in photo‐ or electrochemical conversion areas suggests they are a cost‐effective and superior alternative to the prevalent carbon materials, like graphene and carbon nanotubes.

Though some initial successes have been achieved by GD‐based materials in photoelectric conversion and catalysis areas, this field is still in preliminary stage with significant challenges needed to be solved urgently. First, the few‐layer GD or other kinds of GYs possess more advantages than bulk materials, and the large scale fabrication of high‐quality and defect‐free ultrathin GD or other kinds of GYs still remains a challenge. Second, most studies on GD are still in the theoretical stages and some new members in the GY family with different percentage of acetylenic linkages and adjustable mechanical and electronic structures are anticipated to be obtained, such as 6,6,12‐graphyne. Third, the chemical functionalization of GD and other kinds of GYs, doping them with other elements, and hybridization them with other functional materials are still needed to be improved to endow them with desired bandgap structure and fascinating charge mobility, which play a vital role in a variety of electronic and optoelectronic areas. Fourth, to further develop both fundamental study and application process, the in‐depth understanding of the catalytic nature of GD and GD‐based composites is needed, which may be studied by theoretical simulations and in operando techniques, such as X‐ray adsorption spectroscopy or Fourier‐transform infrared spectroscopy. In a word, more efforts are needed to fully exploit the most valuable nature of these new 2D carbon allotropes. We believe that all the challenges and limitations will be overcome, and the bright future is near in the corner.

## Conflict of Interest

The authors declare no conflict of interest.
